# Late development of esophageal stricture following radiation and chemotherapy for small cell carcinoma of the lung: A case report

**DOI:** 10.1186/1757-1626-1-169

**Published:** 2008-09-19

**Authors:** Michael A Zhang, Christina M Trillis

**Affiliations:** 1 Department of Medicine, Louis Stokes Cleveland Veterans Affairs Medical Center, 10701 East Boulevard, Cleveland, OH 44106, USA; 2 Department of Medicine, University Hospitals Case Medical Center, 11100 Euclid Avenue, Cleveland, OH 44106, USA

## Abstract

**Background:**

The development of esophageal stricture is not an uncommon side effect of radiation and chemotherapy for neck and thoracic malignancies. Depending on the study, it may occur anywhere from 2–3 weeks to 4–8 months after therapy. However, chronic late presentations of post-treatment stricture are highly atypical events.

**Case Presentation:**

The authors describe herein an unusual case of a 65 year old male with esophageal stricture presenting as dysphagia and complicated by multiple episodes of aspiration pneumonia four years after chemoradiation treatment for small cell carcinoma of the lung. The patient's symptoms were ameliorated after esophageal dilation with stenting.

**Conclusion:**

Latent esophageal stricture should be suspected in any patient previously treated with radiation and chemotherapy regardless of how long ago the therapy was initiated.

## Background

Dysphagia is a Greek term which means difficulty with eating. There are many causes of dysphagia, ranging from reflux, malignancy, and connective tissue disorders. It is also known to be a common side effect of radiation and chemotherapy. While the acute development of dysphagia following chemoradiation therapy is well known, reports of late presentations are extremely sparse. Here, we present a case report of a patient who developed symptoms of dysphagia from an esophageal stricture a full four years after initial treatment for small cell carcinoma of the lung.

## Case presentation

A 65 year old white male presented with signs of increasingly severe dysphagia of four months duration. He stated that foods become impacted in his throat shortly after eating, with resultant vomiting and regurgitation. This is worse with solids as opposed to liquids and resulted in the loss of 10–15 pounds over the last several months. He noted these symptoms only in association with meals and not with any changes in position. The patient denied having odynophagia, chest pain, abdominal pain, or constitutional symptoms. He was transferred from an outside hospital with an additional diagnosis of aspiration pneumonia, his third episode in recent months. The patient's past medical history was significant for small cell carcinoma of the right lung, treated with 4 cycles Cisplatin, Etoposide and radiation therapy in 2004 and 2005, and GERD. His list of medications included moxifloxacin, omeprazole, simvastatin, loratadine, and albuterol and flunisolide inhalers. The patient also had a 60 pack year history of smoking.

On admission, the patient's vital signs were a temperature of 97.8, a pulse of 98, a respiratory rate of 16, a blood pressure of 90/50, and 95% hemoglobin saturation on 3 L of oxygen. Physical examination was completely unremarkable, with a non-tender, non-swollen neck, an intact gag reflex, and no epigastric tenderness. Laboratory data revealed an elevated WBC of 12.07 k/cmm with an increased ANC of 10.54 k/cmm and 87.3% bands, a decreased hemoglobin of 10.9 g/dL and a hematocrit of 33.4%. The patient's calcium was slightly lowered at 8.1 mg/dL. Urine specific gravity was 1.006. Other labs were within normal limits. An MBS was performed which was nondiagnostic. Chest x-ray revealed a left apical mass-like consolidation with a mild perihilar infiltrate. Although these findings could simply be due to the patient's pneumonia, the history of dysphagia and weight loss prompted further investigation with a CT scan. While no malignancy was detected, there was evidence of chronic aspiration pneumonitis. The patient was placed on ceftriaxone (1 gm IV) and azithromycin (500 mg IV) and was switched to PO Augmentin at 875 mg/day after 3 days on improvement of his pulmonary symptoms. Because it was known that esophageal dysfunction predisposes patients to repeated episodes of aspiration pneumonia, an EGD was performed for further workup. The EGD demonstrated a tortuous esophagus with pooling of secretions. There was mild increased resistance of LES on passage of the scope. This stricture did not appear inflammatory or malignant and was located adjacent to the area previously irradiated four years ago during treatment for the patient's small cell lung carcinoma.

## Discussion

Here, we present the case of a patient who developed late symptoms of increasingly severe dysphagia probably caused by esophageal stricture secondary to radiation and/or chemotherapy for a prior thoracic malignancy. This is not an entirely uncommon side effect of cancer treatment. In a randomized trial of 51 patients, Kaasa et al reported that 64% of (non) small cell lung cancer patients treated with radiotherapy experienced dysphagia compared to 8% of the chemotherapy patients 6 weeks after the start of treatment [1]. The same agents (cisplatin and etoposide) were used in this study. What is unusual about this case is the fact that the patient presented with dysphagia a full four years after therapy. According to DR Camidge, "symptomatic acute radiation oesophagitis usually develops 2 or 3 weeks after the beginning of treatment and may last for several months" [2]. In the Kaasa study, only 22% of patients still experienced dysphagia eight weeks after treatment. A literature search revealed that chronic esophageal disease after radiotherapy is a rare event. Lawson et al reported the frequency of stricture to be 2.6% and that of stenosis 0.8% following a 60-Gy dose [3]. This concurs with the assessment of Kaplinski et al: "The incidence of late radiation injury of the esophagus is not precisely determined but, overall, the occurrence of clinically apparent damage is infrequent" [4].

There have been few isolated reports on the development of chronic strictures. One paper noted the occurrence of complete esophageal obstruction in a 21 year-old after 14 years of chemo and [thoracic] radiation therapy for Hodgkin's lymphoma [4]. Another study of five children concluded that radiation induced strictures may present up to ten years after therapy [5]. It was hypothesized that children are particularly prone to latent effects given "that organ development and growth may not be complete at the time of treatment." This obviously cannot be the case in adults. What then, might be the cause of this patient's late presentations of post-radiation dysphagia? One possibility is that his stricture could have been secondary to an unrelated process such as GERD or hiatal hernia; in these cases the fact that it was located adjacent to the site of radiation treatment might simply be a coincidence. However, given this patient's known prior history of GERD, it may well be that an exaggeration of reflux was superimposed on a previously subclinical stricture secondary to radiation therapy to produce overt dysphagia. In fact, proton pump inhibitors and sucralfate are known treatments for both GERD and radiation induced strictures [6].

## Conclusion

It is important to identify signs of dysphagia, not only because of the potentially serious causes, including malignancy, but because of the effects on the patient. Here, the development of multiple episodes of aspiration pneumonia is certainly not an insignificant concern. In a report by Pauloski et al., it was noted that patients with dysphagia had a higher rate of aspiration (22% vs. 3%) compared to those who did not after radiation treatment [7]. Six of 29 patients in another study developed aspiration pneumonia after chemoradiation for locally advanced head-and-neck cancer, two of whom later died [8]. In the presenting patient, esophageal dilation was eventually performed with the aim of reducing the aforementioned outcomes. It is quite unfortunate that his symptoms were not recognized earlier. Although it is not particularly common, iatrogenic causes of esophageal stricture should be suspected in any patient with clinical signs of dysphagia status post radiation and/or chemotherapy.

## Abbreviations

ANC: absolute neutrophil count; CT: computerized tomography; EGD: esophagogastroduodenoscopy; GERD: gastroesophageal reflux disease; MBS: modified barium swallow; PO: by mouth; WBC: white blood cell count

## Competing interests

The authors declare that they have no competing interests.

## Authors' contributions

MZ conceived the study and was the principal author of the manuscript. CT was responsible for the care of the patient and contributed to the writing of the preliminary draft. All authors read and approved the final manuscript.

## Consent

Written informed consent was obtained from the patient for publication of this case report. A copy of the written consent is available for review by the Editor-in-Chief of this journal.
